# Supplementary material for: Taxonomy-aware, sequence similarity ranking reliably predicts phage–host relationships
Source: BMC Biol. 2021 Oct 8;19:223. doi: 10.1186/s12915-021-01146-6 (PMC8501573; doi:10.1186/s12915-021-01146-6)
Supplement: Supplementary file 1 — Additional file 1: Figure S1. Discriminatory power of Phirbo, BLAST, and WIsH scores to differentiate between interacting and non-interacting virus-prokaryote pairs. Virus-host pairs were obtained from a. Edwards et al. and b. Galiez et al. data sets. Box plots show the distribution of scores for all interacting virus-host pairs (n = 16,757 and n = 26,024 in Edwards et al. and Galiez et al., respectively) and the same number of randomly selected, non-interacting virus-host pairs. The horizontal line in each box displays the median; boxes display the first and third quartiles; whiskers depict lowest and highest non-outlier scores (details of distributions including outliers are provided in Additional file 2: Table S1). Figure S2. Host predictions for Cronobacter phage ENT39118 (RefSeq accession: NC_019934) using a. BLAST and b. Phirbo. Querying the Cronobacter phage sequence with a BLAST search against the host database returned the genomic sequence of Escherichia coli (NC_017641) as the best match (bit-score = 14,588), and Cronobacter sakazakii (NC_009778) as the second-best match (bit-score = 14,020). Phirbo predicted Cronobacter sakazakii as the top-score host for the Cronobacter phage due to the highest extent of overlap between the top-ranking BLAST matches of each sequence (NC_019934 and NC_009778) of the same database. For clarity, only the first ten BLAST matches are shown. Figure S3. Host prediction performance of Phirbo, BLAST and WIsH over virus contig length in terms of a. Area under the curve (AUC) and b. Area under the precision-recall curve (AUPR). Bars indicate the AUC or AUPR averaged across 10 replicates at a given subsampling length of phage sequence. Figure S4. Scatter plot of the phage sequence coverage used in host predictions of Phirbo versus that of BLAST. Each dot represents a phage genome. Figure S5. Distribution of different bacteria taxa (from species to phylum) across the first 10 depths of BLAST lists obtained from querying a. 820 phage [file 12915_2021_1146_MOESM1_ESM.pdf]

Suplementarny Information for

Zielezinski A, Barylski J, Karlowski WM.

Taxonomy-aware, sequence similarity ranking reliably predicts phage-host relationships.

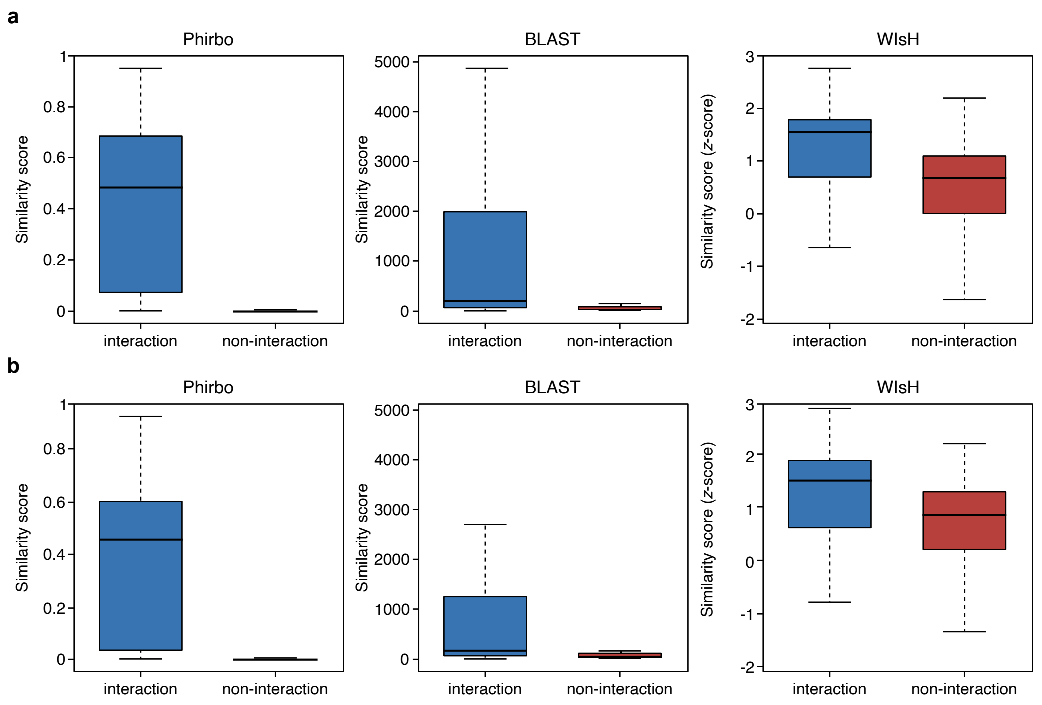

Figure S1. Discriminatory power of Phirbo, BLAST, and WIsH scores to differentiate between interacting and non-interacting virus-prokaryote pairs. Virus-host pairs were obtained from **a.** Edwards *et al*. and **b.** Galiez *et al*. data sets. Box plots show the distribution of scores for all interacting virus-host pairs (n = 16,757 and n = 26,024 in Edwards *et al.* and Galiez *et al.*, respectively) and the same number of randomly selected, non-interacting virus-host pairs. The horizontal line in each box displays the median; boxes display the first and third quartiles; whiskers depict lowest and highest non-outlier scores (details of distributions including outliers are provided in **Additional file 2: Table S1**).

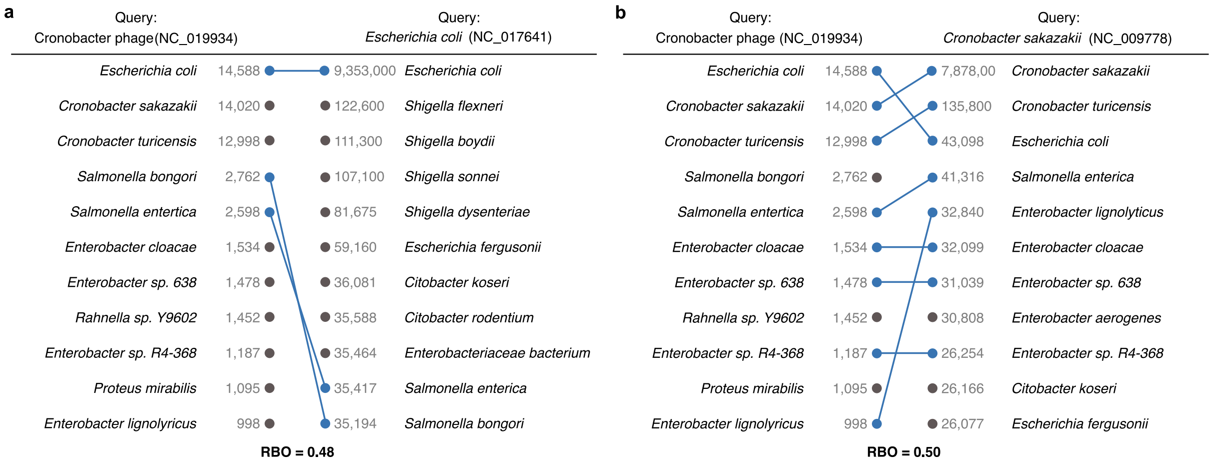

Figure S2. Host predictions for Cronobacter phage ENT39118 (RefSeq accession: NC_019934) using **a.** BLAST and **b.** Phirbo. Querying the Cronobacter phage sequence with a BLAST search against the host database returned the genomic sequence of *Escherichia coli* (NC_017641) as the best match (bit-score = 14,588), and *Cronobacter sakazakii* (NC_009778) as the second-best match (bit-score = 14,020). Phirbo predicted *Cronobacter sakazakii* as the top-score host for the Cronobacter phage due to the highest extent of overlap between the top-ranking BLAST matches of each sequence (NC_019934 and NC_009778) of the same database. For clarity, only the first ten BLAST matches are shown.

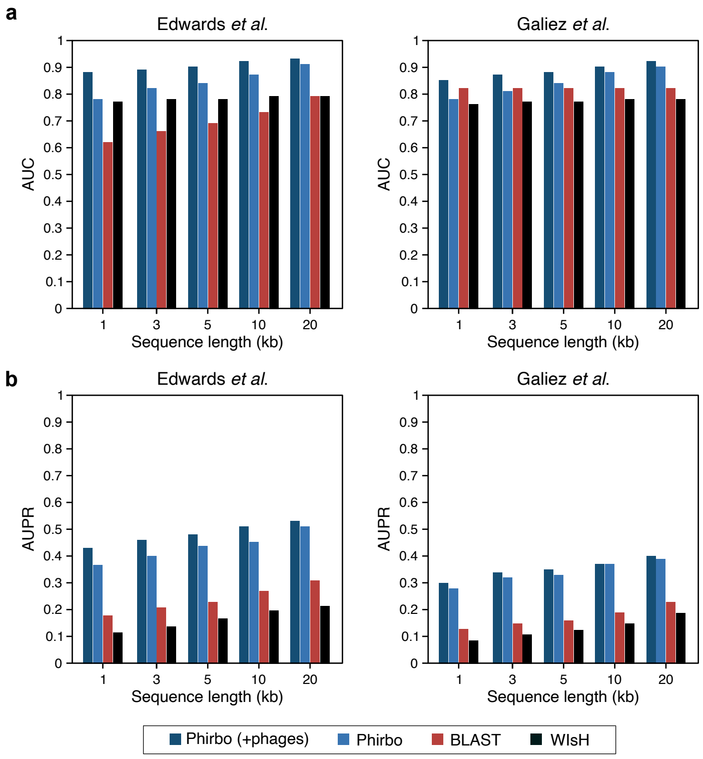

Figure S3. Host prediction performance of Phirbo, BLAST and WIsH over virus contig length in terms of **a.** Area under the curve (AUC) and **b.** Area under the precision-recall curve (AUPR). Bars indicate the AUC or AUPR averaged across 10 replicates at a given subsampling length of phage sequence.

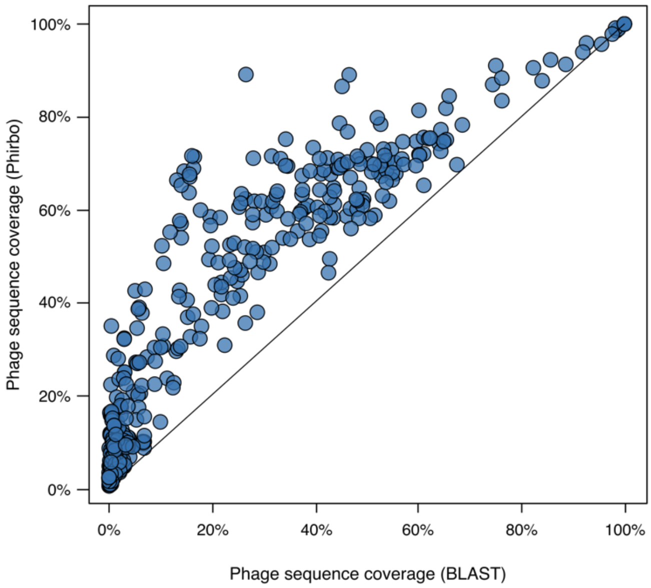

Figure S4. Scatter plot of the viral sequence coverage used in host predictions of Phirbo versus that of BLAST. Each dot represents a phage genome.

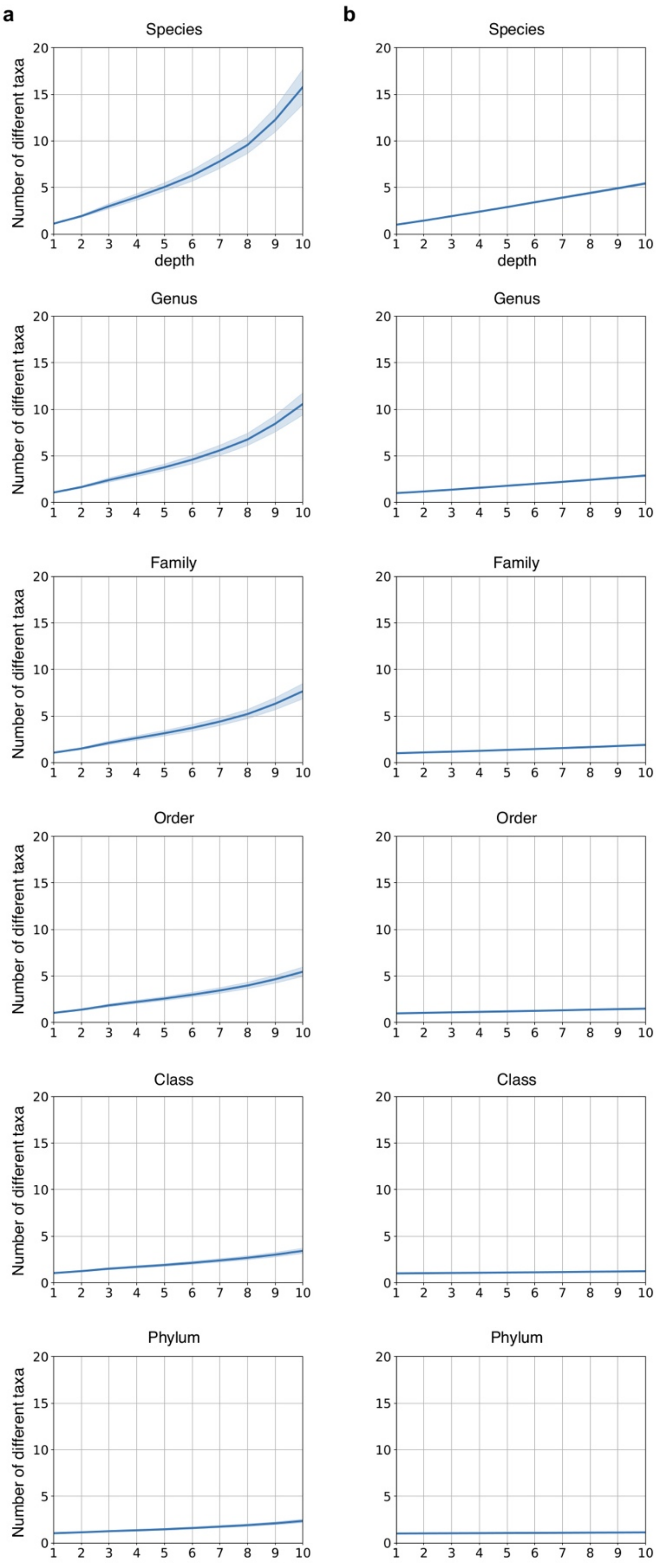

Figure S5. Distribution of different bacteria taxa (from species to phylum) across the first 10 depths of BLAST lists obtained from querying **a.** 820 phage genomes and **b.** 2,699 bacterial genomes from Edwards *et al*. (2016) against a database of the bacterial genomes. The blue line shows the mean of different taxa and the light blue shade indicates the 95% confidence level.

For example, on average there are 10 different bacteria species present in BLAST list up to the 8 ranking (depth = 8).
